# Vagus nerve stimulation in dementia: A scoping review of clinical and pre-clinical studies

**DOI:** 10.3934/Neuroscience.2024024

**Published:** 2024-09-27

**Authors:** Ronald Kamoga, Godfrey Zari Rukundo, Samuel Kalungi, Wilson Adriko, Gladys Nakidde, Celestino Obua, Johnes Obongoloch, Amadi Ogonda Ihunwo

**Affiliations:** 1 Department of Anatomy, Mbarara University of Science and Technology, P.O. Box 1410, Mbarara, Uganda; 2 Department of Psychiatry, Mbarara University of Science and Technology, P.O.Box 1410, Mbarara, Uganda; 3 Makerere University, School of health sciences, Department of Pathology. Kampala, Uganda; 4 Library department, Mbarara University of Science and Technology, P.O. Box 1410, Mbarara Uganda; 5 Faculty of Nursing and Health Sciences, Bishop Stuart University, Mbarara, Uganda; 6 Department of Pharmacology, Mbarara University of Science and Technology, P.O.Box 1410, Mbarara, Uganda; 7 Department of Biomedical engineering, Mbarara University of Science and Technology, P.O.Box 1410, Mbarara, Uganda; 8 University of the Witwatersrand, School of Anatomical Sciences, Faculty of Health Sciences, Johannesburg, South Africa

**Keywords:** cognitive impairment, Dementia, Alzheimer's disease, vagus nerve stimulation, brain stimulation

## Abstract

**Background:**

Dementia is a prevalent, progressive, neurodegenerative condition with multifactorial causes. Due to the lack of effective pharmaceutical treatments for dementia, there are growing clinical and research interests in using vagus nerve stimulation (VNS) as a potential non-pharmacological therapy for dementia. However, the extent of the research volume and nature into the effects of VNS on dementia is not well understood. This study aimed to examine the extent and nature of research activities in relation to the use of VNS in dementia and disseminate research findings for the potential utility in dementia care.

**Methods:**

We performed a scoping review of literature searches in PubMed, HINARI, Google Scholar, and the Cochrane databases from 1980 to November 30th, 2023, including the reference lists of the identified studies. The following search terms were utilized: brain stimulation, dementia, Alzheimer's disease, vagal stimulation, memory loss, Deme*, cognit*, VNS, and Cranial nerve stimulation. The included studies met the following conditions: primary research articles pertaining to both humans and animals for both longitudinal and cross-sectional study designs and published in English from January 1st, 1980, to November 30th, 2023; investigated VNS in either dementia or cognitive impairment; and were not case studies, conference proceedings/abstracts, commentaries, or ordinary review papers.

**Findings and conclusions:**

We identified 8062 articles, and after screening for eligibility (sequentially by titles, abstracts and full text reading, and duplicate removal), 10 studies were included in the review. All the studies included in this literature review were conducted over the last three decades in high-income geographical regions (i.e., Europe, the United States, the United Kingdom, and China), with the majority of them (7/10) being performed in humans. The main reported outcomes of VNS in the dementia cases were enhanced cognitive functions, an increased functional connectivity of various brain regions involved in learning and memory, microglial structural modifications from neurodestructive to neuroprotective configurations, a reduction of cerebral spinal fluid tau-proteins, and significant evoked brain tissue potentials that could be utilized to diagnose neurodegenerative disorders. The study outcomes highlight the potential for VNS to be used as a non-pharmacological therapy for cognitive impairment in dementia-related diseases such as Alzheimer's disease.

## 1. Introduction

Dementia is a group of neurodegenerative disorders characterized by features of cognitive impairment such as memory loss, impaired judgment, and reduced ability to carry out activities of daily living, among others [1],[2]. There are several causes of dementia; however, Alzheimer disease (AD), which is a condition characterized by Amyloid plaques and neurofibrillary tangles in the brain, is the most common cause of dementia in older people, accounting for approximately 60–70% of cases [3],[4]. Due to the lack of effective treatments against Alzheimer's disease and a number of related dementias (ADRDs), there is growing clinical and research interests in using vagus nerve stimulation (VNS) as a potential non-pharmacological therapy for ADRDs [5],[6]. VNS is a Food and Drug Administration (FDA) approved therapy to manage drug resistant epilepsy, major depression, cluster headaches, and inflammatory bowel disease [7]–[10]. There are a number of clinical and preclinical studies that have demonstrated VNS to be associated with reduced neuroinflammation, an increased neuroplasticity, and an improved cognitive performance, though it is mainly in non-cognitively impaired populations [11]–[14]. The scope and extent of research on the effect of VNS in cognitively impaired populations is scanty. Moreover, a number of studies about the effects of VNS in dementia have reported inconsistent outcomes, including either the absence of an effect or the worsening of cognitive functions [3],[15]–[20]. The contradictory findings about the effects of VNS in dementia could be a consequence of the differences in the methods of investigation, the stimulation protocols, and the researcher's knowledge and skills, among others. Thus, due to a growing interest to use VNS to manage various diseases including dementia [21], and the variability of the study results, there is an urgent need to summarize the available literature about VNS in dementia so as to decide the direction of future research and to guide policies and practices with regard to the use of VNS as a potential therapy for dementia. Therefore, this scoping review was conducted to systematically map the research that has been performed, to identify the nature of the existing literature, to summarize and disseminate findings for the potential utility in dementia care policies, and to practice and research guidelines. The review process was guided by the following question: what is known from the existing literature about the use of VNS in dementia or cognitive impairment?

## 2. Methods and materials

### 2.1. Protocol and registration

The protocol for this scoping review was drafted using the Preferred Reporting Items for Systematic Reviews and Meta analyses—extension for scoping reviews (PRISMA-ScR) [22],[23], and was revised by the team. However, the protocol is not publicly available, though it can be accessed on request from the corresponding author.

### 2.2. Eligibility criteria

Only primary articles pertaining to both humans and animals were included for both longitudinal and cross-sectional study designs. All English-language articles about VNS in dementia that had been published from 1^st^ January, 1980 to November, 30^th^ 2023, were included. The start date of 1980 was chosen because research activities regarding VNS as a potential therapy for various conditions were very scanty before 1980 [24], and because the approval and use of VNS as a therapy for various neurological conditions is relatively recent [25]. All articles in languages other than English were excluded because of the cost and time constraints in translating the material. All published articles on VNS in conditions other than AD and case studies, conference proceedings/abstracts, commentaries, and review papers were excluded. We only used studies with readily available full articles. Abstract-only articles were also included. All study designs were represented in order within the review article to obtain as in-depth and broad results as possible.

### 2.3. Information sources and search strategy

In order to identify potentially relevant literature, the following electronic databases were searched from 1980 to 30^th^ November 2023: PubMed, HINARI, Google Scholar, and Cochrane. databases. The following keywords were used to search the various databases: Brain stimulation, dementia, Alzheimer's disease, vagal stimulation, memory loss, Deme*, cognit*, VNS, and Cranial nerve stimulation. The search strategies and the definition of key concepts were developed from the research question with the guidance of a qualified librarian (WA), who helped us to identify the relevant keywords and who advised us on what databases were most likely to produce the type of studies we sought. Additionally, the librarian devised the initial search strategy, which was later refined in light of the early results. However, some databases such as Scopus, EMBSAE, and Web of science were inaccessible due to the lack of subscriptions. The final search results were exported into EndNote, and duplicates were removed by the research team. The Endnote reference manager software was used due to its compatibility with the word processing package we were using, and it was relatively quick and easy to use to generate lists of references to include in the final literature review report. The electronic database search was supplemented by manually searching the reference lists/bibliographies of the identified studies through the database searches, and by scanning relevant reviews and grey literature to ensure they had been included in the scoping exercise. The search strategy and databases that were used are summarized in Table 1.

### 2.4. Data charting process

A standardized data extraction form was jointly developed by three reviewers to ensure that all relevant data based on the study objectives were captured. Then, each of the three reviewers independently screened and charted all the eligible articles by title. After the title screening, the three reviewers compared their results. Any disagreements were resolved through discussions and consultations with a content expert (GZR), who is a senior consultant psychiatrist. Then, the reviewers independently reviewed the eligible studies by reading through the abstracts and charting. They subsequently compared their results, and any disagreements were resolved as previously described. Next, they each independently read the full articles of the eligible studies and charted the data. Finally, they compared the results and resolved the differences as previously described. The data extraction form was continuously updated in an iterative process. A matrix table was used to display the data, and all articles excluded based on the full text review were recorded and the reasons for exclusion were documented.

### 2.5. Variables to be collected

The following variables were collected and recorded for the analysis: the study design, the sample sizes, the type of intervention (invasive or non-invasive or both), the study outcomes (histomorpholgical, molecular, and behavioral outcomes), the year when the data was collected, the geographic location/region/country where the study was performed, socio-demographics for the clinical studies (e.g., age, gender, religion, ethnicity, etc.), the authors' names, the year of publication, the stimulation site, and the stimulation parameters (Table 2).

### 2.6. Quality assessment

A risk of bias across the studies and a critical appraisal of the individual sources of evidence were not performed because the purpose of this scoping review was to provide a general overview of the extent and nature of the existing evidence pertaining to use of VNS in either dementia or cognitive impairment, regardless of the methodological quality or risk of bias [27],[28].

### 2.7. Data analysis

Following the data extraction, a thematic analysis was performed whereby the studies were organized according to the investigated study variables. The key elements of each study were noted, including the study design, the sample sizes, the type of intervention (invasive or non-invasive or both), study outcomes (wanted and unwanted outcomes), the year when the data was collected, the geographic location/region/country where the study was conducted, and the socio-demographics (e.g., age, gender, religion, ethnicity, etc.) used in the participant selection.

## 3. Results

We identified 8062 articles through the database search. After removing 408 duplicates, 7654 articles remained, of which 7346 articles were eliminated because they did not fit the inclusion criteria based on the title screening. Of the remaining 308 articles, 289 articles were excluded after screening the abstracts due to the following reasons: not meeting the inclusion criteria (n = 278); duplicated (n = 8); and protocols/ongoing clinical trials (n = 3). Of the remaining 19 articles, 9 were excluded after the full text screening due to the following reasons: ongoing trials/protocols (n = 3); duplicated (n = 1); a failure to access either the full article or relevant information from abstract (n = 4); and one article was a conference proceeding (n = 1). The figure below illustrates the iterative process of the article selection. Having read the articles in full, 8 articles were selected for inclusion in the review.

**Figure 1.**
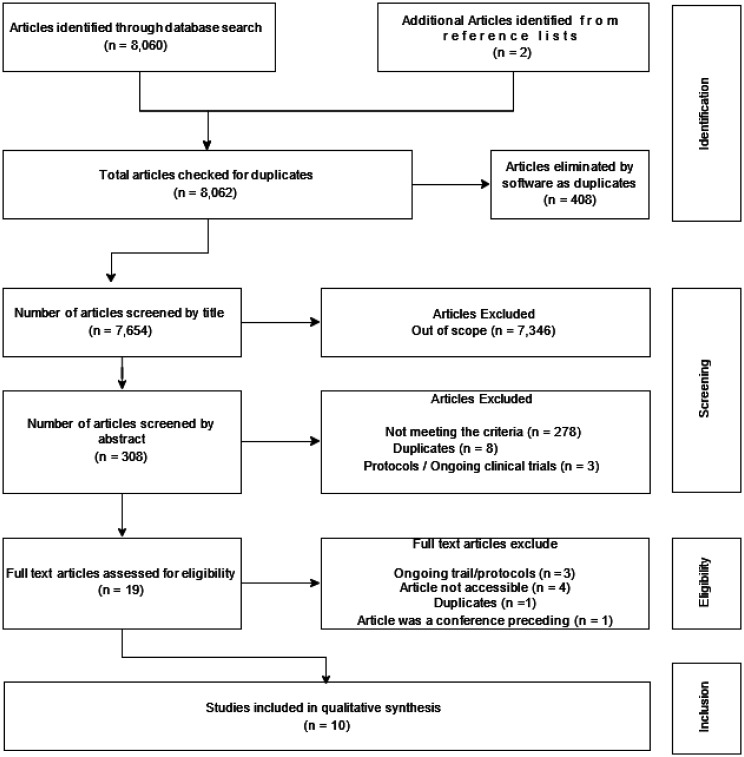
Article selection flow chart.

**Table 1.** Summary of the data bases, search strings and the number of articles obtained from each data base.

**Database**	**Search strategy**	**Number of articles obtained**	**Comments**
PubMed	(((((Vagus nerve stimulation) OR (VNS)) OR (vagal stimulation)) OR (cranial nerve stimulation)) OR (brain stimulation) AND ((((((dementia) OR (Alzheimer's disease)) OR (cognitive impairment)) OR (Memory loss)) OR (deme*)) OR (cogniti*) )))	819 articles	Filters included: meta-analysis; randomized controlled trials; Review; systematic review; 1980/1/1:2023/11/30; English
Cochrane	"vagus nerve stimulation" OR "Vagal stimulation" OR "VNS" OR "Cranial nerve stimulation" OR "Brain stimulation" in Title Abstract Keyword OR "dementia" OR "Alzheimer's" OR "cognitive impairment" OR "memory loss" OR (NEXTdeme*) OR (NEXT cognit*)	5744	5722 were Cochrane systematic reviews while 22 were clinical trials
Google scholar	("vagus nerve stimulation" OR "VNS" OR "vagal stimulation"OR"Cranial nerve stimulation") AND ("Alzheimer's disease" OR "Alzheimer disease" OR "Cognitive impairment" OR "memory loss OR deme* OR cognit*")	980	The search returned 10,900 English results. However, there was a limitation of how many results can be downloaded. The first 980 articles sorted according to relevance were obtained.
Hinari	((vagus nerve stimulation) OR (vagal stimulation) OR (vns) OR (cranial nerve stimulation) OR (brain stimulation)) AND ((alzheimer's) OR (dementia) OR (cognitive impairment) OR (memory loss) OR (deme*) OR (cognit*))	517	Additional filters included; Open access, Full text online, Scholarly and peer reviewed, Journal articles, Publications, 1990 to 2023, English Fields searched included; medicine, biology, pharmacology, therapeutics, zoology, occupational therapy, rehabilitation, neurosciences, neurology, nerve stimulation studies.

**Table 2.** Electronic data extraction form showing the extracted variable from the selected studies.

**Tudy tile**	**Author(s), year/Country**	**Type of VNS intervention/Study deign**	**Type of study/Gender/ age range**	**Sample size**	**Stimulation parameters: sites; invasive/non-invasive stimulation; intensity; frequency and duration/duty cycles and timing in relation to learning stimulation**	**Outcomes/Conclusions**
1. The mechanism underlying chronic transcutaneous auricular vagus nerve stimulation in patients with mild cognitive impairment through the enhancement of the functional connectivity between the left precuneus and parahippocampus gyrus	Wang et al., 2023/ China [29]	Noninvasive/Randomized Controlled Trial (RCT)	Clinical study/Males& females/55-75 years	60 with MCI and 30 healthy control participants	**Sites** • Experimental taVNS group- two auricular acupoints were stimulated, including heart (CO15) and kidney (CO10), in the distribution of vagus nerve. • Sham taVNS group- another two auricular acupoints were stimulated, including elbow (SF3) and shoulder (SF4, 5), out of the distribution of vagus nerve. **Stimulation** • taVNS group, a pulse train of 20 Hz for 10 s and 100 Hz for 50s in each minute, intensity from 0.6 mA to 1.0 mA, determined by individual tolerance of the patients, 30 min in each session, two sessions every day, once in the morning, the other in the afternoon or evening, five consecutive days per week with an interval of 2 days for rest, treatment period for 24 weeks	• Increased functional connectivity (FC) between the left medial prefrontal lobe and right lingual gyrus at the baseline in patients with MCI. • Declined FC between the left/right hippocampus and middle/upper frontal gyrus, and between the left/right precuneus and parahippocampal gyrus. • Only the FC between the left precuneus and parahippocampus was enhanced, marginally positive correlated with the overall score of MoCA-B and AVLT-N7, after 24 weeks of taVNS • There was no other reverse regulation of the FC between each pair of brain regions within the DMN. • taVNS can improve cognition in patients with MCI through enhancing the FC between the left precuneus and parahippocampus.
2. The efficacy and safety of transcutaneous auricular vagus nerve stimulation in patients with mild cognitive impairment: A double blinded randomized clinical trial	Wang et al., 2022/ China [30]	Noninvasive/Randomized Controlled Trial (RCT)	Clinical study/Males& females/55-75 years	60	**Site** • In taVNS group, a pair of auricular acupoints were stimulated, including heart (concha, CO15) and kidney (CO10), in the distribution of vagus nerve. • In sham VNS group, another pair of auricular acupoints were stimulated, including elbow (scaphoid fossa, SF3) and shoulder (SF4,5), out of the distribution of vagus nerve. **Stimulation** • A pulse train of 20 Hz for 10s and 100Hz for 50s in each minute, intensity from 0.6 Ma to 1.0 mA, determined by individual tolerance of the patients, 30 min in each session, two sessions every day, once in the morning, the other in the afternoon or evening, five consecutive days per week with an interval of 2 days for rest, treatment period for 24 weeks.	• Significant difference in the overall scores of MoCA-B between taVNS group and sham taVNS group (p = 0.033 < 0.05). • In taVNS group, compared with those before intervention, the overall scores of MOCA-B increased significantly after intervention (p < 0.001); and in sham taVNS group, compared with those before intervention, there was no significant difference in the overall scores of MoCA-B after intervention (p = 0.338). • Compared with sham taVNS, there was also significant difference in the difference value of pre- and post-intervention in taVNS group (p < 0.001). • For immediate recall, there was significant increase in N5 post-intervention compared to pre-intervention (p < 0.001) within the taVNS group; and no significant difference in N5 post-intervention compared to pre-intervention (p = 0.059) within the sham taVNS group. • For delayed recall, Pre-intervention: No significant difference between taVNS and sham taVNS groups (p = 0.470). Post intervention: No significant difference between taVNS and sham taVNS groups (p = 0.056). Significant increase in N7 post-intervention compared to pre-intervention (p < 0.001) within taVNS group. No significant difference in N7 post-intervention compared to pre-intervention (p = 0.051) within sham taVNS group • Significant difference in the change from pre- to post-intervention between taVNS and sham taVNS groups (p = 0.005).
3. Vagus Somatosensory Evoked Potentials (VSEPs) – A Possibility for Diagnostic Improvement in Patients with Mild Cognitive Impairment?	Metzger et al., 2012/ German[31]	Noninvasive/ Randomized Controlled Trial (RCT)	Clinical study/Males& females/ Mean age 73.5 ± 8.4 years for AD group, mean age 69.5 ± 5.0 years for MCI group, mean age 70.1 ± 5.7 years for healthy controls	51 participants (12 patients with AD, 12 patients with MCI, 27 healthy subjects)	**Site** • Right and left tragus **Stimulation** • Electrical square impulses of 0.1 ms duration, 8 mA intensity, and a frequency of 0.5 Hz were applied at the left and right tragus in separate trials. The electrical brain activity was recorded with a sampling rate of 20 KHz, a band-pass of 0.1–1 KHz, and an epoch length of 10 ms separately for right and left stimulation.	• Identifiable evoked potentials noted during stimulation at the right tragus with increasing latencies from healthy controls to mild cognitive impairment, indicating the potential diagnostic value of VSEPs in Alzheimer's disease.
4. The effects of transcutaneous vagus nerve stimulation on functional connectivity within semantic and hippocampal networks in mild cognitive impairment	Murphy and Aidan, 2023/USA[32]	Noninvasive/ Randomized Controlled Trial (RCT)	Clinical study/Females/ Average age 75	50	**Sites** • Experimental group- Left tragus-left auricular branch of the vagus nerve • Control group- electrodes were placed on opposite sides (mesial and lateral faces) of the earlobe. • The return electrode for tVNS was placed anterior to the tragus to minimize off-target stimulation, and the sham return electrode was placed on the mesial face of the ear lobe. **Stimulation** • Positive pulses were delivered at a 20 Hz, 50 µs pulse. • Stimulation was delivered continuously during one fMRI resting state condition. • Stimulus intensity for sham and tVNS was progressively increased from 0 to the threshold of discomfort, then reduced to 80% of threshold, as per prior investigations. Due to device limitations, stimulation intensity was capped at 10 mA, which most subjects reached without discomfort.	• During unilateral left taVNS, compared with ear lobe stimulation, patients with MCI showed alterations in functional connectivity between regions of the brain critical for semantic and salience functions including regions of the temporal and parietal lobes. Furthermore, connectivity from hippocampi to several cortical and subcortical clusters of ROIs also demonstrated change with tVNS compared with ear lobe stimulation. • In conclusion, tVNS modified the activity of brain networks in which disruption correlates with worsening in Alzheimer's disease.
5. P3-032 Effects of vagus nerve stimulation on cognition, CSF-Tau and cerebral blood flow in patients with Alzheimer's disease: results of a 1 year pilot study	Merrill & Bunker 2004 [33]/USA	iVNS/Single arm follow-up study	n=15	Clinical study	Stimulation parameters inaccessible	• Improvement or no decline from baseline on the ADAS-cog score in 33% and on the MMSE in 64% of participants. • Significant improvement from baseline was sustained at 6 months for the ADAS (median improvement of 3 points, p = 0.011, n = 16) and MMSE (median improvement of 2 points, p = 0.012, n = 16). • After 12 months of VNS, the median change in CSF-tau was a reduction of 7.7% (p = 0.003, n = 15) • Conclusion: a positive effect of VNS on cognition in Alzheimer's disease after one year of treatment
6. Microglia modulation through external vagus nerve stimulation in a murine model of Alzheimer's disease	Kaczmarczyk et al., 2017/German (Europe) [34].	Noninvasive/ Experimental study	Male/female/ 6 month old mice to 12 month old mice	Pre-clinical study	**Site** • Stimulation was performed 13mm anterior to the neck's base and 2mm from the trachea over the vagus nerve. **Stimulation** • The mouse was stimulated twice for two minutes with a three-minute break in between. • The signal consisted of 1 ms duration bursts of 5 kHz sine waves, repeated at 25 Hz. • The signal amplitude was increased until there was strong muscle stimulation, corresponding to approximately 1.8 mA. After the stimulation or sham-treatment, the same three regions were imaged five times. • Sham treatment was performed in the same manner as the nVNS only without turning on the stimulator.	• Significant changes in microglial morphology were dependent on stimulation parameters were noted (p = 0.001). • The effects of nVNS were slightly but significantly different between young and old animals (p = 0.024) • 12-Month-Old APP/PS1 Mice: nVNS caused a significant increase in the number of branches compared to sham-treated controls, with an increase of 3.82 ± 0.90 branches every 40 minutes (p < 0.001) • 12-Month-Old Wild Type (WT) Mice: There was a non-significant trend showing an increase of 2.07 ± 0.88 branches every 40 minutes (p = 0.071). • 6-Month-Old Mice (Both Groups): No significant differences in microglial morphology between nVNS and sham-treated mice. • tVNS significantly associated with morphological changes related to a neuroprotective phenotype in microglia
7. Improvements in associative memory and spatial navigation with acute transcutaneous Vagus Nerve Stimulation in Mild Cognitive Impairment: preliminary data.	Dolphin et al., 2023[36]./ Ireland, United Kingdom	Non-invasive/Experimental study	n=28; Mean age 71.5 ( range 55-85) years; 17M/11F;	Clinical study/	The mean stimulation time pre-cognitive assessments was 21.2 minutes, with mean amplitude setting during active stimulation of 2.5 mA (1.8–4.5) and sham of 2.0 mA (0.9–3.1).	• During FNAT, active tVNS had no effect on facial recognition or reaction times, however recall accuracy was significantly improved (69.2% ±3.13) compared to baseline (44.7% ±3.51 p = 0.016) and sham (50.1% ±3.28 p = 0.021) and during active tVNS spatial navigation (38.94 sec [±1.68]) was quicker than baseline (51.49 sec (±3.2) p = 0.0164) and sham (51.9 sec (±3.15) p = 0 0.0038) • noted no significant improvements in SART or other cognitive tests performance during tVNS
8. Transcutaneous Vagus Nerve Stimulation Effects on Functional Connectivity of the Hippocampus in Mild Cognitive Impairment.	O'Neal et al., 2023[37]/ USA	Non-invansive/ Experimental study	n=50(sham n=25, experimental n=25); F28/M22; age range 60-89 years.	Clinical study	• Stimulation parameters inaccessible	• Contrasting tVNS and sham stimulation, whole-brain seed-to-voxel analysis demonstrated significant changes in connectivity from the left hippocampus to several cortical and subcortical regions bilaterally. • Increased connectivity between the hippocampus and the prefrontal regions and cingulate gyri, and decreased connectivity to anterior and medial temporal lobes. • A seed-to-voxel analysis from the right hippocampus indicated significant decrease in connectivity to a single cluster of regions in the left anterior temporal lobe in response to tVNS. • tVNS modified connectivity from the hippocampus to multiple brain regions involved in learning and understanding, which disruption correlates with deterioration in AD
9. Vagus nerve stimulation in patients with Alzheimer's disease: additional follow-up results of a pilot study through 1 year	Merrill et al., 2006 [39]/ Europe (Sweden)	iVNS / Longitudinal follow-up study	Clinical study	n= 17	Stimulation parameters not accessible	• Improvement or no decline from baseline of 41.2% and 70.6% on the ADAS-cog and MMSE, respectively after 1 year. • Twelve of 17 patients were rated as having no change or some improvement from baseline on the Clinician Interview-Based Impression of Change (CIBIC+)
10. Cognition-enhancing effect of vagus nerve stimulation in patients with Alzheimer's disease: a pilot study	Sjogren et al., 2002[40]/ Europe (Netherlands)	iVNS/ Longitudinal follow-up study	Clinical study	n =10	Stimulation parameters inaccessible	• After 3 months of treatment, 7 of 10 patients were responders according to the ADAS-cog (median improvement of 3.0 points), and 9 of 10 patients were responders according to the MMSE (median improvement of 1.5 points). • After 6 months of treatment, 7 patients were responders on the ADAS-cog (median improvement of 2.5 points), and 7 patients were responders on the MMSE (median improvement of 2.5 points).

Legend: AVLT-H- auditory verbal learning test-HuaShan version; MOCA-B - Montreal cognitive assessmentbasic; MCI- mild cognitive impairment; CO- Concha ; taVNS- transcutaneous auricular vagus nerve stimulation; VNS- vagus nerve stimulation; SF- scaphoid fossa; DMN- default-mode network; Nvns – Noninvasive vagus nerve stimulation; VSEPs - Vagus Somatosensory Evoked Potentials; FNAT- Face-Name Association Task; SART- Sustained Attention Response Test; MADRS- Montgomery-Asberg Depression Rating Scale; MMSE-Mini Mental State Examination; ADAS-cog -Alzheimer Disease Assessment Scale, cognitive section.

### 3.1. Study geographical location and study designs

Of the 10 studies included, two studies [29],[30] were conducted in China, four studies [34] were conducted in Europe [39]–[42], one study was conducted in the United Kingdom [36], and three studies were conducted in the United States [33],[37],[43]. Regarding the study designs, there were 9 clinical studies, four Randomized Controlled Clinical Trials (RCTs), two experimental studies, and three longitudinal follow-up studies. There was one pre-clinical experimental study that involved a murine model of Alzheimer's disease. Of the clinical studies, 3 involved people with Alzheimer's disease, while 6 involved people with a mild cognitive impairment. Most studies (7/10) utilized the non-invasive VNS (nVNS) technique.

### 3.2. Stimulation sites and parameters

Non-invasive vagus stimulation was trans-auricular [29],[30], except in one study, where it was within the trans-cervical region [34]. For invasive stimulations, implantation was performed in the cervical region at 13 mm anterior to the neck's base and 2 mm from the trachea. The stimulation parameters varied from study to study, with current frequencies between 0.5 Hz to 100 Hz and intensities from 0.6 mA to 4.5 mA, depending on the participants' tolerance.

### 3.3. Study outcomes

In general, nVNS was associated with an increased functional connectivity in the brain regions that are critical for cognitive functions [26],[29], an improved immediate and delayed memory/cognition among people with mild-cognitive impairments (MCI) [30] and significant microglial structural changes [34].

## 4. Discussion

This scoping review aimed to examine the extent and nature of research activities in relation to the use of VNS in dementia and to summarize and disseminate the research findings. All the studies included in this literature review were conducted over the last three decades in high-income geographical regions (i.e., Europe, the United States, the United Kingdom, and China), with the majority of them (7/10) being performed in humans. The main reported outcomes of VNS in the dementia cases were enhanced cognitive functions, an increased functional connectivity of various brain regions involved in learning and memory, microglial structural modifications from neurodestructive to neuroprotective configurations, a reduction of cerebral spinal fluid tau-proteins, and significant evoked brain tissue potentials that could be utilized to diagnose neurodegenerative disorders.

All studies identified for inclusion in this review were conducted in high-income countries. This may be due to the absence of necessary competencies such as surgical skills to perform invasive VNS procedures, or financial limitations that may prevent access to VNS devices. Additionally, despite the higher prevalence of dementia in lower-income countries, the estimated costs of dementia are substantially higher in higher-income countries [44], thus making dementia research a higher health-care priority in those countries. Lower income nations appear to have lower medical, non-medical, and indirect costs associated with dementia compared to higher income countries, which is most likely due to disparities in services and cultural views of ageing and dementia [45]–[47]. For instance, lower income countries have lower dementia diagnostic coverages, possibly due to a healthcare workers' lack of knowledge and skills in dementia assessments and diagnoses, as well as the influence of cultural beliefs that attribute signs of dementia to either aging or witchcraft [48]. These factors can lead to missed diagnoses, which consequently minimize the disease burden and the estimated costs of dementia, as well as the need for prioritizing dementia research in low-income countries [44]. Moreover, in lower-income economies, the sick elderly are typically cared for by family members within their homes, and it is this indirect home-based care that is projected to be the major driver of dementia costs in higher-income countries [44]. The aforementioned factors may explain why dementia research has received less attention in lower-income countries. Additionally, we noted that all the identified studies were conducted among human subjects except for one study that evaluated the effects of VNS on the structure of microglia in a rat model of Alzheimer's disease [34]. The financial burden associated with animal studies could be the major reason for the limited number of animal studies identified, especially in relation to the preparation of transgenic animal models of cognitive impairment [49]. Furthermore, because VNS is already an established treatment for a number of human diseases, obtaining ethical clearance to conduct research is much easier [50]. Moreover, because animal models have shorter life spans, they may not faithfully mimic all of the time-dependent pathophysiological changes of a human disease that develop over extended periods of time such as Alzheimer's disease [51].

We further noted that 70% (7/10) of the studies included in this review utilized non-invasive (trans-cutaneous) electrical vagus stimulation (tVNS). The greater preference for tVNS was most probably due to its better safety profile since it does not involve surgery; hence, surgery related complications can drive up the expenses [25],[52],[53]. There are two approaches of VNS: 1) Invasive VNS (iVNS), in which a pulse generator is implanted beneath the skin in the upper chest under the clavicle, and the cuff electrodes are connected to the left cervical vagal nerve[25],[40],[54]–[57]; and 2) the tVNS, which involves transcutaneously stimulating the vagus nerve through the auricular branches of either the vagus nerve (atVNS) or the cervical vagus nerves (ctVNS) [58]–[60]. Research has shown that between 4 to 30% of patients who undergo iVNS experience unfavorable side effects that may necessitate repeat surgeries for correction [25],[61]. These side effects may include hematomas, infections, vocal cord paralysis, hoarseness, parasthesias (tingling sensations in the neck region), a shortness of breath, spontaneous turnoff, lead breakage, and stimulator malfunctions, among other things [60]–[62]. Both iVNS and tVNS have been reported to be equally effective [2],[63]–[67], although other studies have found differences in which specific psychophysiological responses were absent in tVNS compared to iVNS [68],[69]. This indicates that tVNS may have weaker benefits when compared to iVNS, most likely because tVNS does not directly stimulate the vagus nerve, which is located deep within the carotid sheath of the neck.

Regarding outcomes of electrical VNS in dementia, the following VNS effects were reported: an enhanced cognitive function, a functional connectivity between different brain areas, a reduced cerebral spinal fluid tau-proteins, neuroprotective microglial structural changes, and neuroprotective morphological changes [33],[36],[39],[40],[74],[75]. Similar results have been reported in related studies conducted in various disease conditions other than dementia [25],[37],[43],[76]–[78], in healthy volunteers [38],[79],[80], and in animal models [35],[38],[81],[82]. The mechanism by which VNS affects cognitive functions is not fully understood; however, according to prior research, VNS is associated with increased levels of Norepinephrine (NE) and neutrophins, specifically, the fibroblast growth factor (FGF-1) and the brain-derived neurotrophic factor (BDNF), within the cerebral cortex, the amygdala, and the hippocampus [83]–[85]. BDNF and FGF-1 reportedly promote enhanced cell signaling through interactions with p75 cellular receptors and tyrosine kinase receptors, as well as a reversal of synaptic loss among other functions, thus resulting in long-term potentiation (LTP), which is a key mechanism of learning and memory [62],[86],[87]. These effects may account for the enhanced cognition and functional connectivity reported in this study. Moreover, NE lowers the inflammatory gene expression in glial cells, thereby lowering the glial expression of pro-inflammatory molecules such as cytokines, tumor necrosis factor, and cell adhesion molecules [88]. Furthermore, decreased levels of NE are associated with microglial structural changes collectively called dystrophic microglia, including decreased branches, cellular shrinkage, and an increased stroma volume that makes the cell more oval-shaped [89]. These changes reduce the microglial motility and phagocytic functions, and hence reduce the ability of the microglia to protect neurons against foreign agents [88],[90]. When dystrophic microglia fail to destroy foreign bodies, they remain in a state of chronic activation and produce excessive proinflammatory molecules, which begin to destroy synaptic connections, and hence weaken the neuronal functioning and cause neuronal death [25],[89], which is a characteristic feature of neurodegenerative diseases. Therefore, it inhibits the release of proinflammatory molecules by dystrophic microglia because VNS increases the release of NE into the brain [83]–[85]; thus, it may be protective against neuronal loss. Additionally, VNS has been associated with an increased microglial length and microglial branching [41], as well as a reduction in the amounts of cerebral spinal fluid-tau proteins [33]. The transformation of the neurodestructive dystrophic microglial structures to a neuroprotective structural configuration, as well as a reduction in the CSF concentration of tau-proteins, which are proteins implicated in the pathogenesis of many neurodegenerative diseases, may suggest that VNS could potentially be used in the treatment of neurodegenerative conditions. However, the aforementioned effects of VNS related to structural changes and the consequent improvement of cognitive functions have not always been reproducible. For instance, there are numerous studies where VNS did not have any effect on cognition in general or in specific areas of cognitive abilities [62],[91]–[94]. The inconsistent outcomes could be due to variations in the stimulation protocols. For instance, it has been demonstrated that the electrical VNS for memory enhancement exhibits an inverted U-shaped curve, wherein a stimulation at a moderate intensity of 0.4 mA was linked to memory enhancement, while no or very little positive effect was seen at lower intensities such as 0.2 or higher intensities such as 0.8 mA [60],[95].

### 4.1. Strengths and limitations

There were some limitations to this review study. First, we only reviewed research published in English. The English language barrier may have resulted in the exclusion of several potentially relevant studies. However, a greater diversity in our publication sample could have provided a broader perspective on the use of VNS in dementia. Second, as a scoping review, we did not assess the methodological quality of the included research reports; therefore, we did not ascertain whether specific studies produced robust or generalizable results. This should be considered when interpreting the findings. However, the effects of VNS on cognitive function observed in multiple investigations were generally consistent, thus lending a degree of credibility to the findings. Furthermore, we worked hard to adequately document the review process so that future investigators might replicate the study, thus increasing both the reliability of the findings and the methodological rigor [96],[97]. This scoping review identified and summarized studies on the use of VNS in cognitive impairments based on the volume, nature, and characteristics of the original research that can be used in policies, practice, and future investigations.

## 5. Conclusion

The majority of published research on the impact of VNS in dementia have been undertaken on human participants in high-income nations. The overall effect of VNS in dementia is an enhanced cognitive performance, which suggests that it may be an effective non-pharmacological therapy for cognitive impairment in dementia-related diseases such as Alzheimer's disease.

## Use of AI tools declaration

Not applicable.
